# Investigating Potential Chromosomal Rearrangements during Laboratory Culture of *Neisseria gonorrhoeae*

**DOI:** 10.3390/microorganisms6010010

**Published:** 2018-01-20

**Authors:** Russell Spencer-Smith, Simon W. Gould, Madhuri Pulijala, Lori A. S. Snyder

**Affiliations:** 1 School of Life Sciences, Pharmacy, and Chemistry, Kingston University, Penrhyn Road, Kingston upon Thames KT1 2EE, UK; russell.smith@nih.gov (R.S.-S.); S.Gould@kingston.ac.uk (S.W.G.); pretty.madhu@gmail.com (M.P.); 2 National Cancer Institute, National Institutes of Health, 9000 Rockville Pike, Bethesda, MD 20892, USA

**Keywords:** gonococcus, genome rearrangement, prophage, PFGE, pulsed field gel electrophoresis, genome sequence

## Abstract

Comparisons of genome sequence data between different strains and isolates of *Neisseria* spp., such as *Neisseria gonorrhoeae*, reveal that over the evolutionary history of these organisms, large scale chromosomal rearrangements have occurred. Factors within the genomes, such as repetitive sequences and prophage, are believed to have contributed to these observations. However, the timescale in which rearrangements occur is not clear, nor whether it might be expected for them to happen in the laboratory. In this study, *N. gonorrhoeae* was repeatedly passaged in the laboratory and assessed for large scale chromosomal rearrangements. Using gonococcal strain NCCP11945, for which there is a complete genome sequence, cultures were passaged for eight weeks in the laboratory. The resulting genomic DNA was assessed using Pulsed Field Gel Electrophoresis, comparing the results to the predicted results from the genome sequence data. Three cultures generated Pulsed Field Gel Electrophoresis patterns that varied from the genomic data and were further investigated for potential chromosomal rearrangements.

## 1. Introduction

*Neisseria gonorrhoeae* is a strict human pathogen and the causative bacteria of the second most common sexually transmitted infection, gonorrhoea. As of 2008, the complete circularized genome sequence of *N. gonorrhoeae* strain NCCP11945, from an infected woman in Korea, became the second publically available gonococcal genome [1]. The availability of this second genome sequence led to interesting comparisons between *N. gonorrhoeae* strains [2,3,4,5,6,7,8,9,10,11,12,13,14,15,16].

Prior to the genomic era, macrorestriction maps of *N. gonorrhoeae* strain FA1090 in comparison with the closely related *Neisseria meningitidis* strain Z2491 showed widespread chromosomal rearrangements between the species [17]. Large scale rearrangements have been shown in silico between genome sequences of *N. gonorrhoeae* strains FA1090 and NCCP11945 [2]. In comparing these two gonococcal strains, inversions and rearrangements were noted, encompassing much of the genome [2]. Within strain rearrangements have also been noted, including a large inversion of approximately 26 kb between pilin variants N137 and N138 of *N. gonorrhoeae* strain MS11, due to recombination between homologous sequences [18].

There are nine identified integrated prophages in *N. gonorrhoeae* strain FA1090, some of which are complete and active [19,20]. There is evidence that ISNgo2 elements within filamentous prophages play a role in large scale genome rearrangements [21]. IS1016 elements also contribute to genome-wide rearrangements between *N. gonorrhoeae* strain FA1090 and *N. meningitidis* strain Z2491 [22]. Comparison between the genome sequences of *N. gonorrhoeae* strains FA1090, TCDC-NG08107, and NCCP11945 identified prophages, IS elements, and neisserial Correia Repeat Enclosed Elements (CREE) repeats as mediating the majority of the differences in genomic synteny [10]. Recombination between three lysogenic phages, NGOФ1, NGOФ2, and NGOФ3, resulted in the inversion and rearrangement of sections of the genome with other rearrangements being associated with ISNgo2 elements in filamentous prophages.

Therefore, it appears that regions of homologous DNA, insertion sequences, and integrated prophages have been major driving forces behind chromosomal rearrangements and major contributors to evolution in *N. gonorrhoeae*. However, this is often based on comparisons between strains and species, investigating the changes in synteny that may have occurred over a relatively long period of evolutionary time. In this study, chromosomal rearrangements were sought after a short period of laboratory culture, in order to determine if the activity of such systems can be seen in currently circulating strains. *N. gonorrhoeae* strain NCCP11945 was continuously and stressfully cultured over an eight week period. The optimum temperature to cause autolysis in *N. gonorrhoeae* is 40 °C [23], so cultures were grown at just below this, 39 °C. DNA that is released from lysed gonococcal cells may be taken up and integrated into surviving cells’ genomes, contributing to potential rearrangements [24]. The accepted time between passages for *N. gonorrhoeae* is 48 h [23,25], and therefore, cultures with an increased passage time, 72 h, may result in genomic arrangements. Growth of bacteria in the presence of nalidixic acid has been shown to induce prophages [26,27], so cultures of *N. gonorrhoeae* with sub-lethal concentrations of nalidixic acid were explored for changes in the genome. Transduction of genetic material by bacteriophages has been demonstrated in *N. gonorrhoeae* and may have a role in chromosomal changes [24]. Resulting start and endpoint cultures were analyzed by restriction digestion and pulsed field gel electrophoresis (PFGE). These results were compared against next-generation genome sequencing data to investigate the potential presence of large genomic rearrangements between cultures. 

## 2. Materials and Methods

### 2.1. Growth Conditions

*N. gonorrhoeae* strain NCCP11945 [1] was cultured on GC agar plates (Oxoid) with 20 μM iron III nitrate and a glucose-based nutritional supplement [25] in a culture jar with CO_2_Gen sachets (Oxoid). GC broth was formulated as previously [28]. Preliminary tests of growth conditions determined that *N. gonorrhoeae* strain NCCP11945 was able to grow at 41 °C, but was unsustainable through repeated passage, and therefore 39 °C was selected as the temperature stress for eight weeks’ passage. Likewise, repeated passage at four days was not sustainable, and therefore three-day passage was used for nutrient stress. Sub-lethal nalidixic acid concentrations were established by growing *N. gonorrhoeae* strain NCCP11945 on GC agar containing 2, 4, 8, 16, 32, 64, 128, and 256 μg/mL nalidixic acid. Although 256 μg/mL nalidixic acid permitted a low level of growth, it was again not sustainable through repeated passages and 128 μg/mL nalidixic acid was used as the prophage inducing stress. Viable cultures under three conditions (39 °C, passage each three days, and with 128 μg/mL nalidixic acid) and a control (37 °C and passage each two days) were maintained in duplicate for a period of eight weeks. At each passage, a sweep of growth on the plates was resuspended in GC broth and used to inoculate the next plate, thereby avoiding potential bias via the selection of individual colonies. Growth was determined visually based on colony size and number observed.

### 2.2. Pulsed Field Gel Electrophoresis

Start-point and end-point samples from the cultures were grown overnight in triplicate and the growth was resuspended in GC broth, embedded in agarose, and DNA extracted, as per the Bio-Rad CHEFII Plug Kit protocol. The agarose plugs were restriction digested with 20 U *Spe*I (Fermentas, Loughborough, UK) for 16 h at 37 °C. *Spe*I digested plugs were loaded onto a 1% agarose gel with flanking PFG lambda size markers (New England Biolabs, Hithchin, UK). The *Spe*I PFGE was run on a Bio-Rad CHEFII pulsed field system at 150 V in 0.5× TBE buffer at 15 °C for 48 h with pulse times ramped from 5 s initially to 120 s final time. *Bgl*II digested plugs were loaded onto a 1% agarose gel with flanking PFG low-range size markers (New England Biolabs, Hitchin, UK) and run on a Bio-Rad (Watford, UK) CHEFII pulsed field rig at 200 V in 0.5× TBE buffer at 15 °C for 24 h with pulse times ramped from 1 s initially to 25 s final time. All gels were stained in a 1 µM solution of ethidium bromide for 45 min, washed in dH_2_O for 45 min, and visualized under UV light.

### 2.3. Predicted PFGE Band Patterns

**Figure 1:**
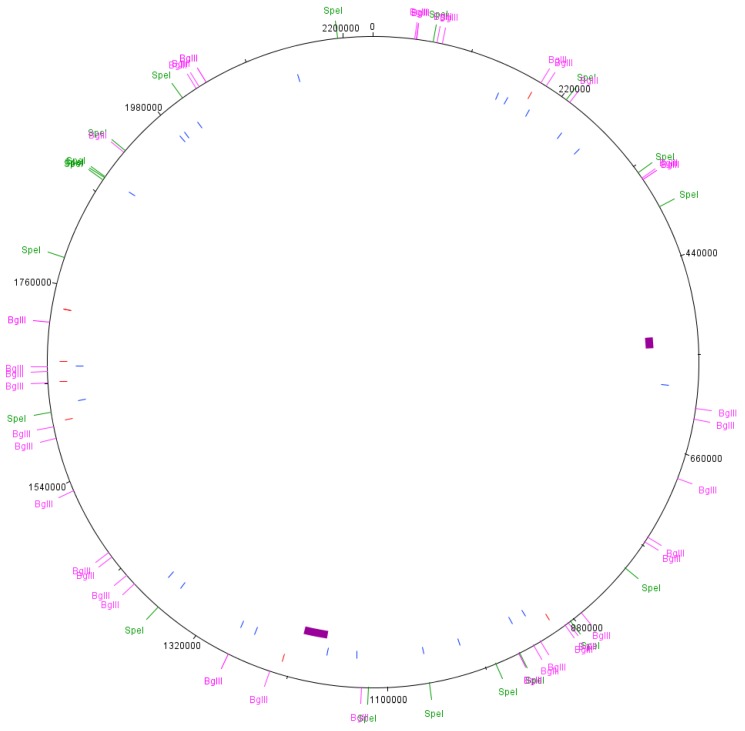
Chromosomal map of *N. gonorrhoeae* strain NCCP11945. Each of the identified restriction digest sites was mapped to the chromosome of *N. gonorrhoeae* strain NCCP11945: *Spe*I shown in green and *Bgl*II in pink. In red are locations of ISNgo2 elements, in blue are IS1016, and purple blocks represent the locations of the NGOΦ1.

The complete genome sequence data from *N. gonorrhoeae* strain NCCP11945 [1] was analysed for the presence of *Spe*I restriction enzyme recognition sites, 5′-ACTAGT-3′ (Table S1), and *Bgl*II restriction enzyme recognition sites, 5′-AGATCT-3′ (Table S2), using the Sequence Manipulation Suite: DNA Pattern Find [29]. These enzymes were chosen based on previous PFGE and mapping experiments in other *N. gonorrhoeae* strains [30,31]. The predicted fragment sizes and their locations in the genome sequence [1] were noted and mapped to the chromosome using DNAPlotter [32] (Figure 1).

### 2.4. Next Generation Genome Sequencing

Cultures were grown for eight weeks under standard conditions and for eight weeks with sub-lethal 128 μg/mL nalidixic acid. DNA extraction used the Gentra Puregene Yeast/Bact. Kit (Qiagen, Manchester, UK); 1 μg of the extracted DNA was genome sequenced on the Life Technologies Ion Personal Genome Machine using the Ion Express Fragment Library kit, Ion Express Template kit, and Ion Sequencing kit (Life Technologies, Loughborough, UK). Generated sequence data was mapped to the reference sequence data (CP001050; [1]) using Galaxy. The Ion Torrent bam format files were converted to fastq format using BAMTools Convert [33]. FASTQ Groomer was used on all NGS data [34]. Bowtie2 was used to map the reads against the reference [35,36] before visualization using the Integrated Genomics Viewer (IGV) [37,38]. Each restriction enzyme digest site was manually analysed for SNPs or other changes to the recognition sequence. Mapped reads were assessed manually in IGV for split reads at suspected chromosomal breakpoints. De novo assembly of unmapped reads used Unicycler [39]. Data is available from NCBI SRA SRR3547950 (standard) and SRR4431963 (nalidixic acid).

## 3. Results

### 3.1. Growth of Bacteria in the Laboratory under Different Conditions

Differences in the growth of the *N. gonorrhoeae* strain NCCP11945 in the four culture conditions (37 °C, 39 °C, three-day passage, 128 μg/mL nalidixic acid) initially resulted in observable differences between the cultures. *N. gonorrhoeae* could not be successfully repeatedly passaged at 41 °C, but was successful at 39 °C. *N. gonorrhoeae* strain NCCP11945 grew at 2, 4, 8, 16, 32, 64, 128, and 256 μg/mL nalidixic acid, but the growth on 256 μg/mL nalidixic acid was not sustainable. Passage each four days likewise resulted in a failure of the culture to grow, whilst passage each three days remained viable. Thus, cultures were propagated for eight weeks under control conditions, 39 °C, three-day passage, and with 128 μg/mL nalidixic acid.

Control cultures grown at 37 °C and passaged to fresh media every two days showed consistent growth throughout the eight-week period. Cultures passaged every three days had approximately 20% more colonies on the third day compared to day two. Although there was more growth on day three than day two, the rate of growth on the plate appeared lower than seen for the previous two days. Initial growth for the 39 °C cultures was approximately 50% fewer colonies than observed in the control cultures; however, the size of individual colonies was larger. Successive passages at 39 °C increased the colony number to a level comparable to or greater than the control. Nalidixic acid containing cultures initially showed approximately 40% fewer colonies, which were approximately 20% smaller than those of the control culture. By week two, the colony size and number was equivalent to that of control cultures.

### 3.2. Pulsed Field Gel Electrophoresis

**Figure 2:**
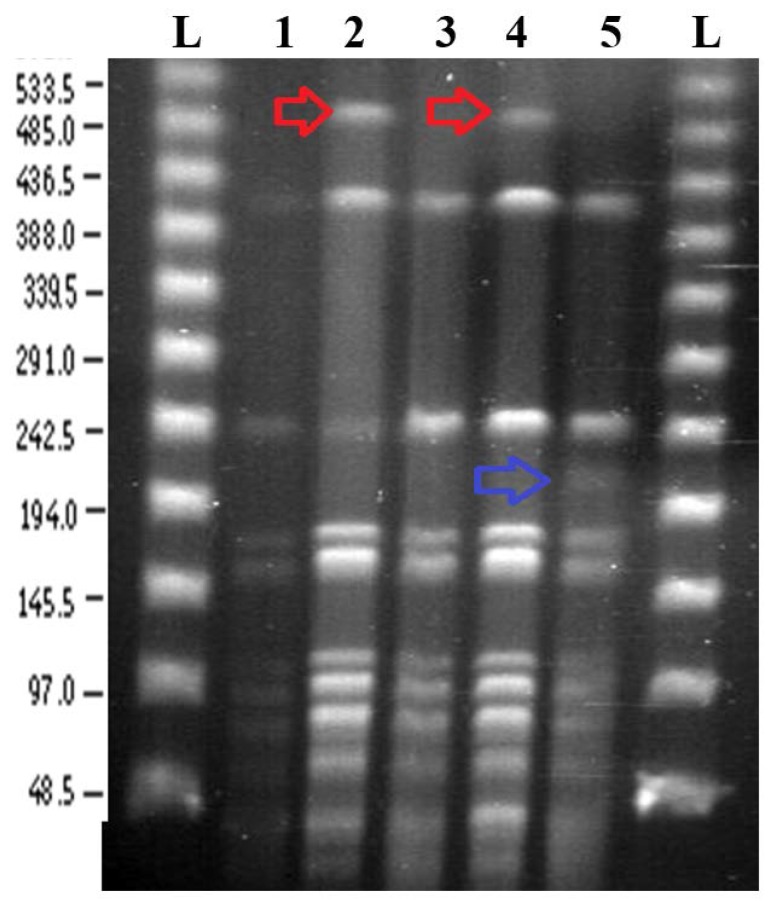
PFGE gel image of *Spe*I digested *N. gonorrhoeae* strain NCCP11945 cultures. L: NEB lambda ladders; 1: temperature stress culture grown at 39 °C for eight weeks; 2: control culture; 3: starting inoculum; 4: nutrient stress culture passaged every three days; 5: prophage stress culture grown with 125 μg/mL nalidixic acid. Coloured arrows represent bands that are present only in those lanes: red arrows, about 500 kb; blue arrow, about 210 kb.

**Figure 3:**
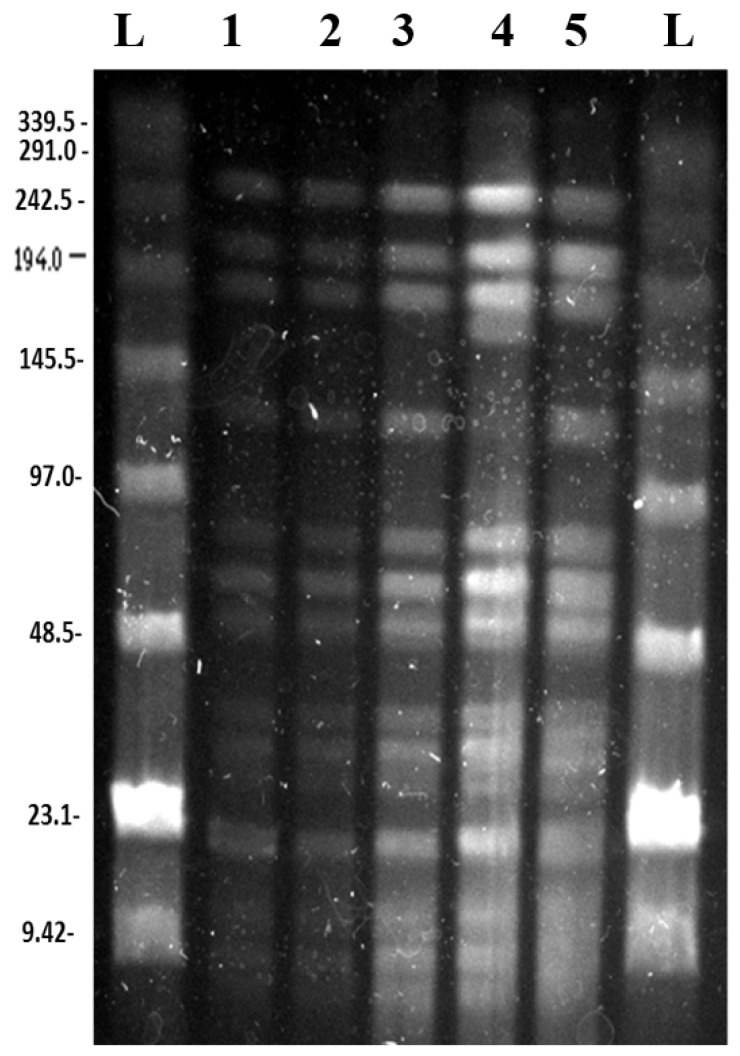
PFGE gel image of *Bgl*II digested *N. gonorrhoeae* strain NCCP11945 cultures. L: NEB lambda ladders; 1: temperature stress culture grown at 39 °C for eight weeks; 2: control culture; 3: starting inoculum; 4: nutrient stress culture passaged every three days; 5: prophage stress culture grown with 125 μg/mL nalidixic acid.

Both SpeI digestion and BglII digestion produced a majority of reproducible bands across all experiments (Figure 2 and Figure 3). These results correlated well with the expected band sizes predicted from the reference genome sequence [1].

#### 3.2.1. *Spe*I Digestion and PFGE

PFGE of *Spe*I digested of agarose plugs containing *N. gonorrhoeae* strain NCCP11945 DNA, produced bands that are present in all of the culture conditions and the starting culture (Figure 2). There is an additional band of approximately 500 kb in the lanes for the control culture and the culture passaged every three days (nutrient stress) that is not evident in the other lanes. The lane for the culture grown on 128 μg/mL nalidixic acid shows an additional band around 210 kb.

#### 3.2.2. *Bgl*II Digestion and PFGE

The PFGE of the *Bgl*II digestions showed bands that are clearly present in all of the lanes, representing the different cultures and their starting inoculum (Figure 3). The culture that was passaged every three days has an additional band of around 168 kb and the almost complete absence of the band of approximately 130 kb (Figure 3).

### 3.3. Predicted PFGE Band Patterns Based on Genome Sequence Data

Based on the whole genome sequence of *N. gonorrhoeae* strain NCCP11945 [1], the restriction digest sites were identified and fragment sizes predicted for *Spe*I (Table S1) and *Bgl*II (Table S2). Comparison between this in silico data and the experimental data (Figure 2 and Figure 3) demonstrates that the assembly of the *N. gonorrhoeae* strain NCCP11945 genome sequence is robust. All of the genome sequence *Spe*I predicted fragment sizes that could be resolved on the PFGE are present and the majority of the *Bgl*II bands are present aside from a region of the gel around 110,000 bp, where no bands are readily visible and *Bgl*II fragments are too small to be resolved. From the *Bgl*II PFGE, it was observed that there was almost a complete absence of the band at approximately 130,000 bp in the nutrient stress culture (Figure 3). This corresponds to the 131,206 bp band predicted from the genome sequence data (Table S2).

### 3.4. Comparison of PFGE Results with Genome Sequence Data from the Cultures

Ion Torrent next generation sequencing of the control and prophage stress cultures was undertaken to determine the cause(s) of the additional bands observed on the *Spe*I PFGE. This data, mapped to the *N. gonorrhoeae* strain NCCP11945 genome sequence (CP001051.1), showed that the *Spe*I and *Bgl*II restriction endonuclease recognition sequences (Tables S1 and S2) have not changed in the cultures. Therefore, differences in the PFGE bands from those expected are not due to mutation of the digest sites.

Focusing on the sequence data from the prophage stress culture, where an additional band was observed in the *Spe*I PFGE, the mapping of the sequence reads to the reference genome did not reveal any large regions of deletion. De novo assembly of the unaligned reads revealed that all unaligned reads were part of the pNGK plasmid present within *N. gonorrhoeae* strain NCCP11945.

## 4. Discussion

Continuous passage of *N. gonorrhoeae* in the laboratory produced some changes in the growth phenotype. Culturing of *N. gonorrhoeae* strain NCCP11945 at 41 °C could not be sustained (data not shown); however, the bacteria were able to adapt to and thrive at 39 °C. This suggests that *N. gonorrhoeae* is able to survive fevers, such as those associated with gonococcal arthritis; however, a lack of prolonged growth at 41 °C is likely the origin of reported success with artificial fever therapy treatments in the past [40].

PFGE from the cultures showed a majority of reproducible bands that correlated to the predicted band sizes from genomic data. An additional 500 kb fragment was seen in the *Spe*I digest of the control and nutrient stress cultures (Figure 2, lanes 2 and 4). A mutation in the restriction site at genomic position 1,371,473 would join the 250,422 bp and 247,231 bp (Table S1) fragments, making a fragment of 497,653 bp. However, genome sequence data from this sample did not indicate a mutation in this restriction site. A large PFGE band of about 500 kb in *Spe*I digests of *N. gonorrhoeae* strain MS11-N198 has been previously reported [30]. It was determined that this *Spe*I digest site becomes resistant to digestion in 10–90% of the gonococcal population [30]. This is consistent with our results for *N. gonorrhoeae* strain NCCP11945 (Figure 2), where the equivalent *Spe*I digest site to the resistant site in strain MS11-N198 is that at position 1,371,473 in strain NCCP11945 (Figure 1). This also explains why the ~500 kb band is not visible in the other PFGE lanes.

A *Spe*I PFGE fragment of around 210 kb is grown on a sub-lethal concentration of nalidixic acid in the culture (Figure 2, lane 5). Unlike the 500 kb *Spe*I fragment, there are no two adjoining *Spe*I fragments which equal around 210 kb. The additional band may therefore be the result of rearrangement. The chromosomes of *N. gonorrhoeae* strain NCCP11945 and strain FA1090 differ in the presence of the Gonococcal Genetic Island [41,42] and in inversions mediated by CREE [2] and SSREE sequences [10]. Inversions or excisions of these elements cannot account for the generation of the 210 kb PFGE band. 

*Spe*I fragment 250,422 bp contains NGOΦ1, NGOΦ2, NGOΦ3, and NGOΦ7 and fragment 247,231 bp contains NGOΦ2, NGOΦ3, and NGOΦ8. Both include ISNgo2 elements, which have been shown to be the driving factor in several large scale rearrangements in *N. gonorrhoeae* [21]. However, inversions or excisions between these or other ISNgo2 elements in the chromosome [10,19] would also not generate the observed 210 kb fragment. The mapped sequence data has lower coverage in the regions containing these prophages, with some regions of up to 200 bases having no coverage. De novo assembly of the genomic data and alignment with the reference genome suggests that there are deletions and rearrangements in these regions, although the presence of multiple copies of the prophage sequences may have generated misassemblies. NGOΦ2 has been shown to be functional [20], and therefore it is possible that a prophage mediated process has caused changes in the *N. gonorrhoeae* genome in the presence of nalidixic acid, known to activate prophage gene expression. The NGOΦ1 within the 250,422 *Spe*I fragment is 30,030 bp, and therefore, the loss of this prophage would result in a band of 220,392 bp (Figure 1). The precise identity of the additional band on the PFGE cannot be determined given the data; however, it is likely to be due to NGOΦ1 or NGOΦ2. There is a high degree of sequence similarity between the NGOΦ1, NGOΦ2, and NGOΦ3 prophages [20], complicating the mapping of sequence reads and the de novo assembly and alignment of sequences against the reference genome. Therefore, there is no solid sequencing data supporting the mobilisation of prophages. The additional 210 kb band in the nalidixic acid culture is either an experimental artefact occurring in just one lane or the result of changes to the genome via the activity of the neisserial prophage. The latter would be expected under these culture conditions; however, any resulting chromosomal rearrangements would need to be determined using an extra-long sequencing read technology, such as the Oxford Nanopore or PacBio systems.

The *Bgl*II PFGE of the nutrient stress culture has an additional band of approximately 168,000 bp and the corresponding almost complete absence of the 131,206 bp band (Figure 3, lane 4). These bands cannot be accounted for through the loss of a *Bgl*II site. Given the sequence results from the control and nalidixic acid-containing culture, sequencing did not clearly elucidate the cause of the additional PFGE bands and the absence of a range of bands on the *Bgl*II PFGE, and the nutrient stress culture was not sequenced.

It is evident in the sequencing data that small changes associated with phase variable changes in repeat tracts and inversion do occur, as have been observed and reported previously [16,43]. The growth culture phenotypes observed, where the stress cultures grow less well than the control initially and then adapt (Tables S1 and S2), are likely to be due to phase variable switching of gene expression. Phase variation is known to be involved in stochastic switching of gene expression that confers an adaptive advantage over the other cells in the culture [16,44,45,46,47]. Therefore, the observed changes in the phenotype and small changes in the genome sequence data concur with previous studies. De novo assembly of the sequence data and alignment with the reference genome suggests that there may be larger scale rearrangements; however, these could be misassemblies and did not correspond to data from mapped reads. Contigs can be generated that suggest that there are large rearrangements, but without long sequence read data such changes cannot be confirmed. 

## 5. Conclusions

Pulsed field gel electrophoresis can confirm the order of assembly of next generation genome sequence data. Using the final circularized assembly of a bacterial genome sequence, the PFGE digest pattern can be predicted and compared to the in silico data. Over time, changes in the genome can occur in the laboratory. Here, we show that *N. gonorrhoeae* strain NCCP11945 was able to grow at 41 °C, to grow after four days of growth, and to grow on media with 256 μg/mL nalidixic acid; however, growth in these conditions was not sustainable. Observable differences in growth resolved by three weeks in conditions of 39 °C, after passage each three days, and in media with 128 μg/mL nalidixic acid. These adaptations are likely to be due to small genetic changes, such as phase variable gene expression [16,43], which are evident in corresponding sequence data. Potential large scale chromosomal rearrangements suggested by additional bands on PFGE are due to a previously reported resistant restriction enzyme digest site [30] and potential filamentous prophage induction and transposition. It is clear that investigations into the genotypic origins of phenotypic changes in *N. gonorrhoeae* are complex, involving phase variable changes, changes in restriction site sensitivity, and rearrangements mediated by prophage and prophage associated elements, all of which may dynamically change in culture, contributing to population diversity.

## Supplementary Materials

The following are available online at http://www.mdpi.com/2076-2607/6/1/10/s1.

Supplementary File 1
