# Supplementary material for: Investigating Potential Chromosomal Rearrangements during Laboratory Culture of Neisseria gonorrhoeae
Source: Microorganisms. 2018 Jan 20;6(1):10. doi: 10.3390/microorganisms6010010 (PMC5874624; doi:10.3390/microorganisms6010010)
Supplement: Supplementary File 1 [file microorganisms-06-00010-s001.pdf]

Table S1. Predicted *Spe*I restriction fragment lengths for *N. gonorrhoeae* strain NCCP11945.

***Spe*I fragment size (bp)**	**Fragment start position[1]**	**Fragment end position[1]**
419,153	381,983	801,136
250,422	1,121,051	1,371,473
247,231	1,371,473	1,618,704
183,210	2,010,254	2,193,464
170,847	1,618,704	1,789,551
160,031	66,148	226,179
111,567	226,179	337,746
104,709	2,193,464	66,148
95,718	1,789,551	1,885,269
85,396	1,924,858	2,010,254
83,451	801,136	884,587
76,063	978,232	1,054,295
66,756	1,054,295	1,121,051
65,045	884,587	949,632
44,237	337,746	381,983
35,803	1,889,055	1,924,858
28,600	949,632	978,232
2,202	1,885,269	1,887,471
1,584	1,887,471	1,889,055

[1] Genomic location of the start and ends of the restriction fragments from CP001050 [1].

Table S2. Predicted *Bgl*II restriction fragment lengths for *N. gonorrhoeae* strain NCCP11945.

*Bgl*II fragment size (bp)	Fragment start position[1]	Fragment end position[1]
261,562	347,126	608,688
236,780	2,041,308	46,065
205,250	1,717,000	1,922,250
177,466	950,923	1,128,389
131,206	1,276,072	1,407,278
116,692	76,236	192,928
114,644	230,582	345,226
106,388	1,922,250	2,028,638
104,457	765,491	869,948
101,717	1,128,389	1,230,106
77,486	1,451,617	1,529,103
71,558	687,865	759,423
66,812	621,053	687,865
60,844	1,529,103	1,589,947
48,382	1,602,474	1,650,856
48,143	1,668,857	1,717,000
45,966	1,230,106	1,276,072
32,083	891,801	923,884
31,213	199,369	230,582
25,036	1,420,272	1,445,308
22,491	48,105	70,596
18,343	932,580	950,923
17,494	869,948	887,442
12,994	1,407,278	1,420,272
12,648	1,650,856	1,663,504
12,527	1,589,947	1,602,474
12,365	608,688	621,053
9,176	2,032,132	2,041,308
8,696	923,884	932,580
6,441	192,928	199,369
6,309	1,445,308	1,451,617
6,068	759,423	765,491
5,640	70,596	76,236
5,353	1,663,504	1,668,857
4,359	887,442	891,801
3,494	2,028,638	2,032,132
1,943	46,065	48,008
1,900	345,226	347,126
97	48,008	48,105

[1] Genomic location of the start and ends of the restriction fragments from CP001050 [1].

References

1. Chung, G.T.; Yoo, J.S.; Oh, H.B.; Lee, Y.S.; Cha, S.H.; Kim, S.J.; Yoo, C.K. Complete genome sequence of *Neisseria gonorrhoeae* NCCP11945. *J. Bacteriol.* **2008**, *190*, 6035–6036.
